# Experimental stress in inflammatory rheumatic diseases: a review of psychophysiological stress responses

**DOI:** 10.1186/ar3016

**Published:** 2010-05-17

**Authors:** Sabine JM de Brouwer, Floris W Kraaimaat, Fred CGJ Sweep, Marjonne CW Creemers, Timothy RDJ Radstake, Antoinette IM van Laarhoven, Piet LCM van Riel, Andrea WM Evers

**Affiliations:** 1 Department of Medical Psychology, Radboud University Nijmegen Medical Centre, P.O. Box 9101, 6500 HB Nijmegen, The Netherlands; 2 Department of Chemical Endocrinology, Radboud University Nijmegen Medical Centre, P.O. Box 9101, 6500 HB Nijmegen, The Netherlands; 3 Department of Rheumatology, Radboud University Nijmegen Medical Centre, P.O. Box 9101, 6500 HB, Nijmegen, The Netherlands

## Abstract

**Introduction:**

Stressful events are thought to contribute to the aetiology, maintenance and exacerbation of rheumatic diseases. Given the growing interest in acute stress responses and disease, this review investigates the impact of real-life experimental psychosocial, cognitive, exercise and sensory stressors on autonomic, neuroendocrine and immune function in patients with inflammatory rheumatic diseases.

**Methods:**

Databases Medline, PsychINFO, Embase, Cinahl and Pubmed were screened for studies (1985 to 2009) investigating physiological stress responses in inflammatory rheumatic diseases. Eighteen articles met the inclusion criteria.

**Results:**

Results suggest that immune function may be altered in response to a stressor; such alterations could contribute to the maintenance or exacerbation of inflammatory rheumatic diseases during stressful events in daily life.

**Conclusions:**

This review emphasizes the need for more experimental research in rheumatic populations with controlled stress paradigms that include a follow-up with multiple evaluation points, simultaneous assessment of different physiological stress systems, and studying factors contributing to specific physiological responses, such as stress appraisal.

## Introduction

Stress is widely recognized as an important risk factor in the aetiology of inflammatory rheumatic diseases [1-5]. An adaptational stress response involves the activation of both the hypothalamus-pituitary-adrenal axis (HPA axis) [6] and the autonomic nervous system (ANS) [7], and both stress axes are thought to communicate bidirectionally with the immune system [7-10]. Because many rheumatic diseases are characterized by immune-mediated joint inflammation, stressful events might contribute to the aetiology, maintenance and exacerbation of rheumatic diseases [11,12]. Recent advances in psychoneuroimmunology have provided insight into the complex mechanisms by which stressors might acutely affect the body's immune system [13-16]. However, little attention has been paid to whether and how different short-term experimental stressors influence the separate pathways of the physiological stress response system (ANS, HPA axis, immune system) in patients with inflammatory rheumatic diseases.

Perception of an external stressful stimulus prompts the activation of various physiological systems that together define the body's stress response, which is aimed at re-establishing homeostasis. The physiological stress response is mainly coordinated by the hypothalamus, with activation of the ANS and the pituitary and adrenal glands (HPA axis) resulting in the release of catecholamines and cortisol, respectively [1,9,17]. These stress hormones, supposedly acting via β- and α-adrenergic as well as glucocorticoid receptors, down-regulate immune and inflammatory processes; however, these processes also influence the central nervous system (CNS) [7,18-20]. Circulating cytokines (for example, tumor necrosis factor α (TNF-α), interleukin (IL)-6 and IL-1) and activated immune cells, markers of inflammation, activate both (intermediates of) the HPA axis and the ANS. Chronically elevated levels of cytokines, as occur during long-term inflammation, might lead to changes in HPA axis and ANS activity [21]. Moreover, the bidirectional relationship between the CNS and immune system implies that the physiological response to real-life stressors could contribute to the pathophysiology of inflammatory diseases [1-5]. How these three systems, the ANS, the HPA axis and the immune system, act in response to a stressful event in rheumatic disorders is not well understood.

Although the laboratory setting is not a *natural *environment, it allows control of key factors in the delivery of stress and observation of its effects and reduces many sources of bias and individual differences [16,22]. The literature on acute psychoneuroimmunological and psychoendocrinological responses to experimental stress in healthy individuals is still increasing. Studies of healthy populations suggest that experimental psychological and physical stressors not only activate the ANS [23] and the HPA axis [24], but also influence the immune system by activating innate immunity, as reflected by increased numbers of natural killer (NK) cells and the production of pro-inflammatory IL-6 [15,16]. Moreover, these different physiological systems (ANS, HPA axis and immune system) seem to work in an interdependent fashion [25].

Despite the possible detrimental physiological effects of stress in patients with inflammatory rheumatic diseases, such as an altered disease course, little is known about acute-phase reactants of experimentally induced stress (both autonomic, neuroendocrine and immune). Reviews of acute physiological stress responses have either focussed on one [16,24] or two [2] stress response systems only (for example, ANS and/or neuroendocrine system), and included either only patients with rheumatoid arthritis [2] or a heterogeneous group of both healthy participants and various patient populations [16]. In addition, studies of the relationship between stress and inflammatory rheumatic diseases have often used experimental stressors that do not necessarily mimic real-life stressors. Different types of time-limited experimental stressors have been identified, namely, physical stressors (autonomic function tests, exercise), physiological stressors (corticotropin-releasing hormone (CRH) and (nor)epinephrine infusions, insulin tolerance test and dexamethasone suppression test) and psychological stressors (cognitive tests, public speaking) [2]. Many studies have investigated the effects of these types of stress on components of the stress response system, such as the ANS or the HPA axis, but external validity of these studies of stress is questionable. The prevalence of cardiovascular dysfunction is high after standard tests of autonomic function [26], such as the Valsalva manoeuvre, deep breathing, orthostatic tests, and sustained handgrip. While these tests may trigger autonomic responses, it is not known whether they activate the stress response system and alter neuroendocrine or immune function. HPA axis function has been investigated extensively by challenging specific parts of the HPA axis by means of infusion of CRH, synthetic glucocorticoids, or cytokines [27,28]. Although alterations in HPA axis responsiveness at a hypothalamic, pituitary or adrenal level have been reported, more subtle changes in HPA functioning have also been suggested to occur [27,28]. While injection studies might shed some light on possible altered neuroendocrine responses, the anti-inflammatory effects of exogenously administered glucocorticoids are not necessarily mirrored by increased secretion of endogenous glucocorticoids in response to a real-life stressor. Thus the question remains to what extent different types of experimental stressors that mimic real-life stressful events (for example, psychological stressors and physical exercise) are able to induce an autonomic, neuroendocrine and immune response in patients with inflammatory rheumatic diseases.

To the best of our knowledge this is the first review to investigate whether and how different experimental stressors mimicking real-life stressful events (psychosocial, cognitive, exercise and sensory stressors) influence physiological responses at the three levels (ANS, HPA-axis, immune system) in patients with prototypic inflammatory rheumatic diseases (for example, rheumatoid arthritis (RA) and systemic lupus erythematosus (SLE).

## Materials and methods

This review is limited to studies involving patients with inflammatory rheumatic diseases who were exposed to a time-limited experimental stressor to assess the autonomic, neuroendocrine, and/or immune responses to stress.

### Search strategy

To identify studies, the electronic bibliographic databases MEDLINE, PsychINFO, Embase, Cinahl and Pubmed databases were searched, using the key words rheum*, (idiopathic or psoriatic) arthr*, *spondylitis*, *sclerosis *and *lupus *in combination with the terms *stress *and either *cortisol *or immun* or *epinephrine *or endocrin* or autonom* or hypothalam* or *HPA*. In addition, reference sections of the articles and review papers were hand-searched for relevant articles on psychological and physical stressors and inflammatory rheumatic diseases.

Inclusion criteria were studies published after 1985 in English peer-reviewed journals; evaluation of an experimental laboratory stress task that induces psychological and/or physical (exercise) stress and/or sensory stress (for example, thermoceptive (cold/heat), visual (light), auditive (noise)) by means of a time-limited experimental stressor; patients diagnosed with systemic inflammatory rheumatic diseases, such as RA, juvenile idiopathic arthritis (JIA), ankylosing spondylitis, systemic sclerosis, or SLE; control group consisting of either healthy participants or participants without a systemic inflammatory rheumatic disease, such as osteoarthritis; use of (neuro)endocrine variables (for example, cortisol levels), ANS variables (for example, heart rate, skin conductance, (nor)epinephrine levels), or immune variables (for example, number of leucocytes or lymphocytes, subsets of lymphocytes, interleukin levels) as outcomes. Exclusion criteria were pharmacological studies involving CRH, glucocorticoids, insulin or (nor)epinephrine infusions; studies evaluating a battery of standard autonomic function tests, which include deep breathing, the Valsalva manoeuvre, posture changes, and sustained handgrip, unless they were part of a paradigm with a psychological and/or exercise and/or sensory stressor [29-33]; assessment of anaerobic threshold, peak oxygen consumption, lactate threshold [34,35], fibrinogen and prothrombin time [36]. If a research group published more than one article on the same experimental study but evaluated different outcome measures, both articles were included in the review [29,30,36,37].

Conclusions were based on (uni- or multivariate) statistics for within-group and/or between-groups differences. If studies reported significant between-group differences in repeated measures ANOVAs, baseline values between groups were assumed to be different, unless stated otherwise. If studies did not report significant (within- or between-) group differences, it was assumed that none were found. If studies did not provide statistical analyses, findings were based on mean (± standard error of the mean (SEM) or standard deviation (SD)) or median values. If those values were ambiguous, no conclusions were drawn.

## Results

### Participants

#### Patient groups

Sixteen studies (18 articles) met the inclusion criteria. The study sample characteristics are summarized in Table 1. Nine studies included patients with RA [29,30,34-42] and seven patients with SLE [31,33,35,40,43-45]. Two of these studies involved both types of patients [35,40]. In addition, one study included patients with JIA [46] and one study included a heterogeneous group of patients with inflammatory arthritis (RA, psoriatic arthritis, ankylosing spondylitis and fibrositis) [32]. Fifteen studies included healthy participants without systemic inflammatory rheumatic disease as control and one study, patients with osteoarthritis [36,37]. In three studies a second control group was added, consisting of either patients with myofascial pain [32], patients with sarcoidosis [43], or participants taking corticosteroids [44]. Most studies have relatively small sample sizes (N = 10 to 20 per group), with even smaller sample sizes (N <10) in two studies [35,40]. In addition, patient samples are probably heterogeneous because, for example, strict exclusion criteria regarding comorbidities are lacking in half of all studies [32,33,35,38,42-44,46]. With regard to diagnostic criteria, one study even included also patients with non-inflammatory arthritis [32]. There is also a high variability in pharmacotherapy profiles, but overall these profiles were well-defined for the various classes of drugs (see Table 1), except in two studies [32,33]. To control for the effects of pharmacotherapy some studies excluded patients using specific medication (for example, corticosteroids [35,38-40,46], opioids [39,41], antidepressants [39,40,45], or adrenoceptor antagonists [29,40,45]), or the intake of medication was regulated prior to and on the day of testing [39,41]. In addition, two studies performed subgroup analyses with patients on different medical regimes [41,45].

**Table 1:** Study sample characteristics

Study	Patient sample	Control sample	Pharmacotherapy of patient sample					
			NSAIDs	DMARDs	Cortisteroids	Biologicals	Other	Pharmacotherapy comments:
Dekkers et al., 2001 [38]	N = 29 RA patients; No exclusion criteria given	N = 30 HC; Age and sex matched; Exclusion: chronic disease, chronic pain, heart problems, hypertension	+	+	-^1^	o	o	^1 ^patients taking oral prednisone (5-15 mg) or corticosteroid injections < 3 months prior to study onset excluded
								
Edwards et al., 2009 [39]	N = 19 RA patients; Exclusion: history of myocardial infarction or cardiovascular disease, peripheral neuropathy, Raynaud syndrome, vasculitis, peripheral vascular disease, other autoimmune or rheumatic disorders, current infection, recent history of substance abuse or dependence, pregnancy, mood or anxiety disorder	N = 21 HC; No age and sex difference with patients; Same exclusion criteria as patients;	+^2^	+	-	+	-^3^	^2 ^24 h prior to study onset no NSAIDs intake; ^3 ^Opioids, antidepressants excluded; Stable medication regime of >1 months
								
Geenen et al., 1996/1998 [29,30]	N = 21 RA patients; Exclusion: other serious diseases	N = 20 HC; Age and sex matched; Exclusion: chronic pain, cardiovascular complaints, chronic diseases	+	+	o	o	-^4^	^4 ^α and β adrenoceptor antagonists excluded (only for autonomic response evaluation)
								
Hinrichsen et al., 1989 [43]	N = 14 SLE patients; No exclusion criteria given	N = 14 HC, N = 12 sarcoidosis patients; Age and sex matched;	o	o	+^5^	o	o	^5 ^24 h prior to study onset no corticosteroid therapy;
								
Hinrichsen et al., 1992 [44]	N = 14 SLE patients; No exclusion criteria given	N = 14 HC, N = 10 HC taking corticosteroids; Age and sex matched	o	o	+^6^	o	o	^6 ^4-10 mg; 24 h prior to study onset no corticosteroid therapy
								
Hogarth et al., 2002 [31]	N = 23 SLE patients; Exclusion: diabetes mellitus, hypertension Remark: some patients have comorbid Sjogren syndrome, Raynaud's phenomenon, history of cerebral lupus	N = 23 HC; Age and sex matched; Exclusion: diabetes mellitus, hypertension	+	+	+^7^	o	-^8^	^7 ^1-30 mg; ^8 ^Antihypertensive medication <1 week prior to study excluded
								
Jscobs et al., 2001 [40]	N = 9 RA patients, N = 7 SLE patients; Exclusion: significant cardiovascular diseases, concurrent infections, history of other autoimmune disorders, drug or alcohol abuse; no exacerbation of disease < 4 weeks	N = 15 HC; Age and sex matched; Same exclusion criteria as patients	o	+	-	-	-^9^	^9 ^adrenoceptor antagonists, antidepressants and benzodiazepines taken < 4 weeks prior to study onset excluded
								
Kurtais et al., 2006 [34]	N = 19 RA patients; Exclusion: severe illness, other general contraindications for graded exercise test	N = 14 HC; Age and sex matched; Inclusion: sedentary Exclusion: chronic systemic disease, chronic pain, contraindication for exercise test	+	+	+^10^	o	o	^10 ^7.5-15 mg;
								
Motivala et al., 2008 [41]	N = 21 RA patients; Exclusion: cardiovascular disease, endocrine-related other immune disorders, acute or chronic infections, pregnancy, current psychiatric mood or anxiety disorder	N = 20 HC; Age and sex matched; Same exclusion criteria as patients	+^11^	+	+^12^	+	-^13^	^11 ^24 h prior to study onset no NSAIDs taken; ^12 ^< 10 mg (> 10 mg excluded); ^13 ^Opioids, oral contraception excluded; Stable medication regime >2 months
								
Palm et al., 1992 [42]	N = 18 RA patients; No exclusion criteria given	N = 14 HC; Age and sex matched; Half of all controls had been hospitalized because of coronary heart disease or hypertension	+	+	+^14^	o	o	^14 ^2,5-10 mg
								
Pawlak et al., 1999 [45]	N = 15 SLE patients (N < 10 for subanalyses: NK cytotoxicity and # β-adrenoceptors); Exclusion: significant cardiovascular diseases, concurrent infections, history of other autoimmune disorders, drug or alcohol abuse	N = 15 HC (N < 10 for subanalyses: NK cytotoxicity and # β-adrenoceptors); Age and sex matched; Same exclusion criteria as patients	o	+	+^15^	o	-^16^	^15 ^5-10 mg; ^16 ^Adrenoceptor antagonists, antidepressants, benzodiazepines taken < 4 week prior to study onset excluded
								
Perry et al., 1989 [32]	N = 19 heterogeneous group of arthritis patients (RA, psoriatic arthritis, ankylosing spondylitis, fibrositis); No exclusion criteria given	N = 38 HC, 17 patients with myofascial pain; No exclusion criteria given	+	o	O	o	+^17^	^17 ^Benzodiazepines, psychotropics, and other medication affecting ANS, etc; 12 h prior to onset study no medication taken known to affect ANS
								
Pool et al., 2004 [35]	N = 7 RA patients, N = 6 SLE patients; No exclusion criteria given	N = 10 HC; HC recruited from hospital staff; No exclusion criteria given	+	+	-	o	-^18^	^18 ^Phenothiazines (e.o. drugs influencing PRL levels) excluded
								
Roupe et al., 2000 [46]	N = 20 JIA patients (N = 15 for in vivo analyses); No exclusion criteria given	N = 20 HC (N = 14 for *in vivo *analyses); Age and sex matched;	+	+	-	o	o	
								
Shalimar et al., 2006 [33]	N = 51 SLE patients; No exclusion criteria given	N = 30 HC; Age and sex matched	o	o	O	o	-^19^	^19 ^Medication known to affect HR, BP excluded
								
Veldhuijzen et al., 2005/2008 [36,37]	N = 21 RA patients; Exclusion: previously confirmed acute coronary syndrome, DM, serious psychiatric diseases	N = 10 OA patients; Same exclusion criteria as patients	+	+	+	+	+^20^	^20 ^Analgesics included; no use of oral contraception

RA = rheumatoid arthritis, SLE = systemic lupus erythematosus, HC = healthy controls, JIA = juvenile idiopathic arthritis, OA = osteoarthritis, DM = diabetes mellitus. + = Included; - = Excluded; o = Not mentioned in article. ANS = autonomic nervous system, BP = blood pressure, DMARDs = disease-modifying antirheumatic drugs, HR = heart rate, NSAIDs = nonsteroidal anti-inflammatory drugs, PRL = prolactin.

### Stress paradigms

The stressors included in this review were experimental manipulations of stressful experiences, either of a psychological, exercise and/or sensory nature, and lasted between one minute and two hours.

#### Psychological stressors

Ten of 16 studies used a psychological stressor, applied for one minute to two hours [29-32,36-38,40,41,43-45].

##### Cognitive stressors

Seven studies used cognitive stressors [29-32,36-38,42,44], namely, the Stroop Color-Word Interference test [29,30,38]; a two-minute cognitive discrimination task [29,30]; the Attention and Concentration Test, Syndrome Short Test, computerized-controlled Reaction Time Task and three parts of the Hamburg Wechsler Adult Intelligence Test (comprehension, digit span, block design) [42,44]; Multiple Choice Word Fluency; and the Benton Visual Memory Scale [44]. Mental arithmetic was assessed in two studies, either with a one- or two-minute serial subtraction task [31,32], or with a two-minute paced auditory serial addition test performed while patients were tilted to a head-up-tilt of 64 degrees [36,37].

##### Psychosocial stressors

Three studies used a psychosocial stressor, namely, a 10-minute public speaking task [40]; the Trier Social Stress Test consisting of a five-minute public speech and a five-minute public mental subtraction task [41]; or a ten-minute public speech (including a five-minute preparation period) on the topic 'Children in today's society' [45].

#### Exercise stressors

Three studies used exercise stressors, namely, a bicycle ergometer task, which involved cycling at a rate of 50 revolutions per minute against a resistance of 12.5 watt for three minutes [38]; or an ergospirometric exercise test on a treadmill to the limit of subject's tolerance for several minutes [34,35].

#### Sensory stressors

Eight studies assessed sensory stressors, most of which used thermal stimuli, usually experienced as painful. Five studies assessed responses to a cold stressor [31,33,38,39,46], in which participants either had to put their hand in a bowl filled with (ice) cold water for several minutes [38,39,46], or endure an ice-pack on their hand [31]. One study did not describe the cold pressor task [33]. Heat pulses [39], an acoustic stress test (several minutes) [42,43] and a pupillary light flash test [32] were also used.

### Outcome measures

Outcome measures were self-reported distress, autonomous nervous system responses, neuroendocrine responses, or immune responses (see Tables 2, 3 and 4).

**Table 2:** Autonomic function in patients with systemic inflammatory rheumatic diseases

Parameter	Studies & patients (N)	Baseline patients vs. controls		Stress reactivity within patients		Stress reactivity patients vs. controls	
Heart rate	[29] 21 RA vs. 20 HC	RA:	No difference [29,36,37,41]	RA:	Increase [29,36,37,41]	RA:	No difference [36,37,41]
	[41] 21 RA vs. 20 HC						Altered (↓) [29]
	[36,37] 21 RA vs. 10 OA	SLE:	No difference [31,45]	SLE:	Increase [45]	SLE:	No difference [31] (cogn.) [45]
	[31] 23 SLE vs. 23 HC				Not reported [31]		Altered (↓) (cold) [31]
	[45] 15 SLE vs. 15 HC	Arthr:	Altered (↑) [32]	Arthr:	Increase [32]	Arthr:	No difference [32]
	[32] 19Arthr vs. 38 HC, 17 MFP						
							
Blood pressure (diastolic/systolic)	[29] 21 RA vs. 20 HC	RA:	No difference [29,36,41]	RA:	Increase [29,36,41]	RA:	No difference [36,41]
	[41] 21 RA vs. 20 HC						Altered (↓) [29]/(↑) [41]
	[36] 21 RA vs. 10 OA	SLE:	No difference [31,45]	SLE:	Increase [45]	SLE:	No difference [31,33,45]
	[31] 23 SLE vs. 23 HC		Not reported [33]		Not reported [31,33]		
	[45] 15 SLE vs. 15 HC						
	[33] 51 SLE vs. 30 HC						
							
Mean arterial pressure (MAP)	[37] 21 RA vs. 10 OA	RA:	No difference	RA:	Increase	RA:	No difference
							
Systemic vascular resistance (SVR)	[37] 21 RA vs. 10 OA	RA:	No difference	RA:	Increase severe subgroup	RA:	Altered (↑) severe subgroup
							
Plasma volume (PV)	[36] 21 RA vs. 10 OA	RA:	No difference	RA:	Decrease	RA:	No difference
							
Cardiac output (CO)	[37] 21 RA vs. 10 OA	RA:	No difference	RA:	No response	RA:	No difference
							
Pre-ejection period (PEP)	[41] 21 RA vs. 20 HC	RA:	No difference	RA:	Decrease	RA:	No difference
							
Plasma catecholamines (nor)epinephrine	[42] 18 RA vs. 14 HC	RA:	No difference [40,42] *	RA:	Increase [40]	RA:	No difference [40,42]
	[40] 9 RA, 7 SLE vs. 15 HC				No response [42]		
	[44] 14 SLE vs. 14 HC, 10 HC	SLE:	No difference [40,45]	SLE:	Increase [40,44,45]	SLE:	No difference [40,45,44] *
	[31] 23 SLE vs. 23 HC		Altered (↓) [31]				
	[45] 15 SLE vs. 15 HC		Not reported [44]				
	[46] 15 JIA vs. 14 HC	JIA:	No difference (NE) [46]	JIA:	Increase (NE)[46]	JIA:	No difference [46]
			Altered (↑) (EPI) [46]		No response (EPI)[46]		
							
Skin conductance (SC)	[29] 21 RA vs. 20 HC	RA:	No difference [29]	RA:	Increase [29]	RA:	Altered (↓) [29]
	[32] 19 Arthr vs. 38 HC, 17 MFP	Arthr:	No difference [32]	Arthr:	Increase [32]	Arthr:	Altered (↑) [32]
							
Pupillary constriction	[32] 19 Arthr vs. 38 HC, 17 MFP	Arthr:	Altered (↓)	Arthr:	Not reported	Arthr:	Altered (↓)

* Findings assumed after inspection of descriptive data.

↑ = altered response pattern is more pronounced compared to a control group; ↓ = altered response pattern is diminished compared to a control group; RA = rheumatoid arthritis, SLE = systemic lupus erythematosus, JIA = juvenile idiopathic arthritis, Arthr = heterogeneous group of arthritis patients, HC = healthy controls, OA = osteoarthritis, MFP = patients with myofascial pain,.., NE = norepinephrine, EPI = epinephrine.

**Table 3:** Neuroendocrine function in patients with systemic inflammatory rheumatic diseases

Parameter	Studies & patients (N)	Baseline patient vs. control		Stress reactivity within patients		Stress reactivity patients vs. controls	
ACTH	[38] 29 RA vs. 30 HC	RA:	No difference [34,38,41]	RA:	Increase [34,41]	RA:	No difference [34,38,41]
	[34] 19 RA vs. 14 HC				Not reported [38]		
	[41] 21 RA vs. 20 HC						
							
Cortisol	[30] 21 RA vs. 20 HC	RA:	No difference [34,35,39,41,42]	RA:	Decrease [34,35,42]	RA:	No difference [30,34,39,41,42]
	[38] 29 RA vs. 30 HC		Altered (↑) [30,38]		Change [30]		Altered (↓) [35,38]
	[39] 19 RA vs. 21 HC		No difference [35]		Increase [39,41]		No difference [44]
	[34] 19 RA vs. 14 HC	SLE:	Altered (↓) [44]	SLE:	Decrease [35]	SLE:	Altered (↓) [35]
	[41] 21 RA vs. 20 HC				No response [44]		
	[42] 18 RA vs. 14 HC						
	[35] 7 RA, 6 SLE vs. 10 HC						
	[44] 14 SLE vs. 14 HC, 10 HC						
							
Growth hormone (GH)	[34] 19 RA vs. 14 HC	RA:	No difference	RA:	Increase	RA:	No difference
							
Insulin-like growth factor (IGF-I)	[34] 19 RA vs. 14 HC	RA:	No difference	RA:	No response	RA:	No difference
							
Prolactin	[35] 7 RA, 6 SLE vs. 10 HC	RA:	No difference	RA:	No response	RA:	Altered (↓)
		SLE:	No difference	SLE:	No response	SLE:	Altered (↓)

↑ = altered response pattern is more pronounced compared to a control group; ↓ = altered response pattern is diminished compared to a control group;

RA = rheumatoid arthritis, SLE = systemic lupus erythematosus, HC = healthy controls, ACTH = adrenocorticotropin hormone,

**Table 4:** Immune function in patients with systemic inflammatory rheumatic diseases

Parameter	Studies & patients (N)	Baseline patient vs. control		Stress reactivity within patients		Stress reactivity patients vs. controls	
Leucocytes	[42] 18 RA vs. 14 HC						
	[36] 21 RA vs. 10 OA	RA:	No difference [36,40,42]	RA:	Increase [36,40,42]	RA:	No difference [36,40,42] *
	[40] 9 RA, 7 SLE vs. 15 HC	SLE:	No difference [43,45,44] *	SLE:	Increase [40,43-45]	SLE:	Altered (↓) [40,43,45]
	[43] 14 SLE vs. 14 HC, 12 SD		Altered (↓) [40]				No difference [44] *
	[44] 14 SLE vs. 14 HC, 10 HC						
	[45] 15 SLE vs. 15 HC						
							
Total lymphocytes	[30] 21 RA vs. 20 HC	RA:	Altered (↓) [30,42]	RA:	Increase [30,40]	RA:	No difference [30,40]
	[42] 18 RA vs. 14 HC		No difference [40]		No response [42]		Altered (↓) [42]
	[40] 9 RA, 7 SLE vs. 15 HC	SLE:	Altered (↓) [40,43,45]	SLE:	Increase [40,45]	SLE:	Altered (↓) [43-45]
	[43] 14 SLE vs. 14 HC, 12 SD		Not reported [44]		No response[43,44]		No difference [40]
	[44] 14 SLE vs. 14 HC, 10 HC						
	[45] 15 SLE vs. 15 HC						
							
Total T cells (CD3+)	[30] 21 RA vs. 20 HC	RA:	No difference [40]	RA:	Increase [40]	RA:	No difference [30,40]
	[40] 9 RA, 7 SLE vs. 15 HC		Altered (↓) [30]		No response [30]		
	[43] 14 SLE vs. 14 HC, 12 SD	SLE:	Altered (↓) [40,45]	SLE:	Increase [40,45]	SLE:	No difference [40,43]
	[44] 14 SLE vs. 14 HC, 10 HC		No difference (%) [43,44] *		No response (%) [43,44]		Altered (↓) [45]/(%) [44]
	[45]15 SLE vs. 15 HC						
							
Helper T cells (CD4+)	[40] 9 RA, 7 SLE vs. 15 HC	RA:	No difference [35,40]	RA:	Increase [40]	RA:	Altered (↑) [35,40]
	[35] 7 RA, 6 SLE vs. 10 HC				Decrease [35]		
	[43] 14 SLE vs. 14 HC, 12 SD	SLE:	Altered (↓) [40,45]	SLE:	No response [40,45]/(%) [44]	SLE:	Altered (↓) [45] (%) [43,44]/(↑)
	[44] 14 SLE vs. 14 HC, 10 HC		No difference [35]/(%) [43]		Decrease [35]/(%) [43]		[35]
	[45] 15 SLE vs. 15 HC		Not reported [44]				No difference [40]
							
Cytotoxic T cells (CD8+)	[40] 9 RA, 7SLE vs. 15 HC	RA:	No difference [35,40]	RA:	Increase [40]	RA:	No difference [40]
	[35] 7 RA, 6 SLE vs. 10 HC				No response [35]		Altered (↓) [35]
	[43] 14 SLE vs. 14 HC, 12 SD	SLE:	Altered (↓)[40,45]	SLE:	Increase [40,45]/(%) [43,44]	SLE:	No difference [40,45,44] *
	[44] 14 SLE vs. 14 HC, 10 HC		No difference [35]/(%) [43]		Decrease [35]		Altered (↓) [35]/(%) [43]
	[45] 15 SLE vs. 15 HC		Not reported [44]				
							
B cells (CD19+)	[30] 21 RA vs. 20 HC	RA:	Altered (↓) [30]	RA:	Increase [30]	RA:	No difference [30]
	[43] 14 SLE vs. 14 HC, 12 SD	SLE:	Altered (↑) (%) [43]	SLE:	Increase (%) [43]	SLE:	Altered (↓) (%) [43,44]
	[44] 14 SLE vs. 14HC, 10HC		No difference (%) [44] *		No response (%) [44]		
							
NK cells (CD56+)	[30] 21 RA vs. 20 HC	RA:	No difference [40]	RA:	Increase [30,40]	RA:	No difference [30,40]
	[40] 9 RA, 7 SLE vs. 15 HC		Altered (↓) [30]				
	[44] 14 SLE vs. 14 HC, 10 HC	SLE:	Altered (↓) [40,45]	SLE:	Increase [40,45]	SLE:	Altered (↓) [40,45]
	[45] 15 SLE vs. 15 HC		Not reported [44]		No response [44]		No difference [44]
							
NK cell cytotoxicity	[40] 9 RA, 7 SLE vs. 15 HC	RA:	No difference [40]	RA:	No response [40]	RA:	Altered (↓) [40]
	[45] 4 SLE vs. 8 HC	SLE:	No difference [40,45]	SLE:	No response [40,45]	SLE:	Altered (↓) [40,45]
							
Cytokines							
IL-6	[39] 19 RA vs. 21 HC	RA:	No difference [40,41]	RA:	No response [40]	RA:	No difference [39-41]
	[41] 21 RA vs. 20 HC		Altered (↑) [39]		Increase [39]		
	[42] 18 RA vs. 14 HC				Decrease (not plasma) [41]		
	[40] 9 RA, 7 SLE vs. 15 HC	SLE:	No difference [40]	SLE:	No response [40]	SLE:	No difference [40]
	[46] 15 JIA vs. 14 HC	JIA:	Altered (↑) [46]	JIA:	Increase [46]	JIA:	Altered (↑) [46]
IL-2	[40] 9 RA, 7 SLE vs. 15 HC	RA:	No difference	RA:	No response	RA:	No difference
		SLE:	No difference	SLE:	No response	SLE:	No difference
IL-4	[38] 29 RA vs. 30 HC	RA:	No difference [38,40]	RA:	No response [40]	RA:	No difference [40]
	[40] 9 RA, 7 SLE vs. 15 HC	SLE:	No difference [40]	SLE:	Increase [40]	SLE:	Altered (↑) [40]
IL-8	[46] 15 JIA vs. 14 HC	JIA:	Altered (↑)	JIA:	No response	JIA:	No difference
IL-10	[40] 9 RA, 7 SLE vs. 15 HC	RA:	Altered (↓) (not intracell.)	RA	No response	RA:	No difference
		SLE:	Altered (↓) (not intracell.)	SLE:	No response	SLE:	No difference
							
IFN-γ	[38] 29 RA vs. 30 HC	RA:	No difference [38]	RA:	No response [40]	RA:	Altered (↑) [40]
	[40] 9 RA, 7 SLE vs. 15 HC		Altered (↓) (not intracell.) [40]				
		SLE:	Altered (↓)(not intracell.) [40]	SLE:	No response [40]	SLE:	Altered (↑) [40]
							
TNF-α	[39] 19 RA vs. 21 HC	RA:	No difference [39,41]	RA:	Increase [39,41]	RA:	Altered (↑) [39,41]
	[41] 21 RA vs. 20 HC						
							
β-adenoceptors	[45] 7 SLE vs. 8 HC	SLE:	No difference	SLE:	No response	SLE:	Altered (↓)
							
β-adrenoceptor sensitivity	[45] 7 SLE vs. 8 HC	SLE:	Altered (↓)	Not assessed		Not assessed	
							
sIL-2 receptor	[42] 18 RA vs. 14 HC	RA:	Altered (↑)	RA:	No response	RA:	No difference
							
C-reactive protein (CRP)	[36] 21 RA vs. 10 OA	RA:	No difference	RA:	Increase	RA:	Altered (↑)

* Findings assumed after inspection of descriptive data.

↑ = altered response pattern is more pronounced compared to a control group; ↓ = altered response pattern is diminished compared to a control group;

RA = rheumatoid arthritis, SLE = systemic lupus erythematosus, JIA = juvenile idiopathic arthritis, HC = healthy controls, OA = osteoarthritis, SD = sarcoidosis patients. IL = interleukin, IFN-γ = interferon-γ, TNF-α = tumor necrosis factor α, sIL-2 receptor = soluble interleukin-2 receptor, intracell. = intracellular interleukin concentration on the single-cell level.

#### Self-reported distress

Stress-induced self-reported distress was assessed in only two of 16 studies [41,45]. Subjective distress levels were either measured on a visual analogue scale (VAS) ranging from 0 to 100 [41] or on a VAS *tension and excitement *together with the State Trait Anxiety Inventory (STAI) [45].

#### Measures of the autonomous nervous system

Eleven studies assessed ANS responses (see Table 2) [29,31-33,37,40-43,45,46], namely, heart rate [29,31,32,37,41,45], diastolic and/or systolic blood pressure [29,31,33,41,45], mean arterial pressure, systemic vascular resistance, plasma volume and cardiac output [36,37], and pre-ejection period (PEP) [41]. Furthermore, plasma (nor)epinephrine levels [31,40,42,44-46] and skin conductance [29,32] were assessed.

#### Endocrine measures

Eight studies assessed neuroendocrine responses (see Table 3) [30,34,35,38,39,41,42,44], namely plasma [30,34,38,41,42,44] or serum [35,39] cortisol levels and plasma ACTH levels [34,38,41]. Two studies measured hormones other than those involved in the HPA axis, namely, plasma growth hormone (GH) and insulin-like growth factor I (IGF-I) levels [34] and serum prolactin levels [35].

#### Immunological measures

Twelve studies assessed immune responses (see Table 4) [30,35,36,38-46], namely, the total number of leucocytes [36,40,42-45], and the number of lymphocytes and/or subsets of lymphocytes, including B cells, T cells and NK cells [30,35,40,42-45]. Two studies assessed NK cell cytotoxicity [40,45]. Six studies reported on cytokine levels [38-42,46], namely, plasma levels of cytokine IL-1β [42], IL-6 [39,41,42] and TNF-α [39], *ex vivo *stimulated mononuclear cell production of IL-2 [40], IL-4 [38,40], IL-6 [40,41,46], IL-8 [46], IL-10 [40], interferon (IFN)-γ [38,40] and TNF-α [41], or intracellular concentrations of IL-4, IL-6, IL-10 and IFN-γ [40]. Other inflammation markers were also assessed, such as C-reactive protein (CRP) [36], the number of β-adrenoceptors on monocytes [45], β-adrenoceptor sensitivity [45], and plasma soluble IL-2 receptor levels [42].

#### Time points of outcome measures

All studies reported baseline values for the outcome measures, and all, except two [32,34], reported a resting period of 3 to 45 minutes. During administration of the experimental stressor, physiological reactivity was either assessed at specific time points (in three studies) [35,41,42], or continuously throughout the stress procedure (only for autonomic measures such as heart rate, blood pressure and/or skin conductance; in four studies) [29,32,37,45]. All studies reported immediate post stress measurements, except two studies that only assessed stress reactivity at 30 minutes [34] or 60 minutes [35] after cessation of the stressor. Eight of 16 studies have one [29,30,36-38,40,45] or more [39,41,46] additional follow-up measurement points, ranging from 5 to 60 minutes after cessation of the stressor.

### Baseline characteristics

Autonomic, neuroendocrine, and immune functions were assessed at baseline in patients with rheumatic disorders and controls. Results for the separate outcome measures are summarized in Tables 2, 3 and 4.

#### ANS variables

ANS function at baseline was not different between patients with rheumatic disorders and controls in most studies. Cardiovascular variables, skin conductance, and catecholamine levels did not differ in nine studies [29,31,36,37,40-42,44-46], whereas three studies reported significantly higher levels of autonomic activity at baseline (heart rate and pupil area [32]; epinephrine levels [46]) or lower levels ((nor)epinephrine [31]) in patients with rheumatic disorders compared with controls (see Table 2).

#### Neuroendocrine variables

Neuroendocrine function at baseline was not significantly different between patients with rheumatic disorders and controls. Five of eight studies reported similar baseline levels of cortisol, ACTH, GH, IGF-I, and prolactin [34,35,39,41,42]. Three studies reported higher levels of cortisol at baseline (in patients with RA) [30,38] or lower levels (in patients with SLE) [44] than in controls (see Table 3). Lower cortisol levels at baseline were also observed in one study in which control subjects were taking corticosteroids [44].

#### Immune variables

Baseline leucocyte counts [36,40,42] were not different between patients with RA and controls. However, two of three studies found patients with RA to have lymphopenia at baseline [30,42]. Levels of cytokines and other inflammatory factors (IL-2, IL-4, IL-6, IFN-γ, TNF-α, and CRP) were similar in patients with RA and controls in five studies [36,38-41]. Three studies reported higher baseline levels of IL-6 [39] and soluble IL-2 receptor [42] and lower basal levels of IL-10 and IFN-γ [40] in patients with RA compared with controls.

Baseline leucocyte counts were not significantly different between patients with SLE and controls in most (three of four) studies [43-45]. One study reported baseline leucopenia in patients with SLE [40]. All studies reported lower lymphocyte counts (including lower subsets of lymphocytes) in patients with SLE than in controls [40,43,45]. Despite this baseline lymphopenia, a higher percentage of B cells (from the total lymphocyte count) was found once [43]. Only one study reported similar numbers of helper T and cytotoxic T cells in patients with SLE and controls [35]. Cytokine (IL-2, IL-4 and IL-6) levels at baseline were not significantly different between patients with SLE and controls [40]. However, cytokine levels of IL-10 and IFN-γ at baseline and the sensitivity of β-adrenoceptors, which are involved in the transduction of autonomic signals to immune cells, were lower in patients with SLE than in controls [45]. The one study with immunological data on patients with JIA reported higher cytokine levels (IL-6 and IL-8) than in controls at baseline [46] (see Table 4).

In summary, at baseline autonomic function is similar in patients with RA and SLE and controls, with only limited evidence for heightened autonomic function in (a subgroup of) patients with rheumatic diseases. Neuroendocrine function at baseline is also comparable in patients with RA and SLE and controls, with only three studies reporting altered cortisol levels in patient groups. Specific immune variables, mainly (subsets of) lymphocyte counts, appear to be lower in patients with SLE than in controls.

### Psychophysiological responses to stress

An overview of findings is given in Tables 2, 3 and 4.

#### Self-reported distress

Self-reported distress was increased significantly from baseline by psychosocial stress in patients with RA [41] and SLE [45] and in controls. This increase was greater in patients with RA [41] than in controls. This was not the case for patients with SLE [45].

#### ANS variables

The autonomic response to stress was assessed in patients with RA, SLE, JIA and in a heterogeneous group of patients with inflammatory arthritis. Results are summarized in Table 2. In patients with RA, experimental stress increased autonomic activity (heart rate [29,36,37,41], blood pressure [29,37,41], systemic vascular resistance [37], pre-ejection period (PEP) [41], plasma volume [36], skin conductance [29], and plasma (nor)epinephrine levels [40]), with the increase being similar to that seen in controls in most studies. However, three studies reported either diminished [29] or more pronounced [37,41] autonomic responses in (a subgroup of) patients. In patients with SLE, experimental stress increased autonomic activity (heart rate [45], blood pressure [45], and (nor)epinephrine levels [40,44,45]), the increase often being similar to that seen in controls [31,33,40,45]. Only one study observed a diminished autonomic response (heart rate), but only during one specific type of stressor (cold) [31]. The one study involving patients with JIA showed experimental stress to increase norepinephrine levels to a similar extent in patients and healthy controls [46]. In one study with a heterogeneous group of patients with inflammatory arthritis, experimental stress increased autonomic activity (heart rate, skin conductance, and pupillary constriction) [32], but this increase was smaller (pupillary constriction) or greater (galvanic response) than that of controls.

In summary, patients with rheumatic disorders respond to experimental stress with increased cardiovascular, galvanic and catecholamine responses. Whereas most autonomic responses to stress are similar to those of a control group, there is partial evidence (five studies) for altered stress-induced autonomic responses.

#### Neuroendocrine variables

Neuroendocrine reactivity, mainly measured as changes in cortisol levels, was assessed in patients with RA and SLE and in controls (see Table 3). Experimental stress elicited both an increase [39,41] and (more often) a decrease [34,35,38,42] in cortisol levels in patients with RA. ACTH, which is activated upstream of cortisol, increased in response to stress [34,41]. In addition to the assessment of HPA axis hormones, one study also reported an increase in GH levels in response to a stressor [34]. The neuroendocrine response to stress of patients with RA was not significantly different from that of controls in most studies, but two studies reported that cortisol responses were diminished in patients with RA compared with controls [35,38]. In one study, experimental stress increased prolactin levels in controls but not in RA patients [35]. The effect of stress on neuroendocrine function in patients with SLE was inconsistent, with stress eliciting a decrease [35] or no change [44] in cortisol levels. A lack of responsiveness was also reported in control subjects [35,44]. Experimental stress did not increase prolactin levels in patients with SLE [35].

In summary, although cortisol and ACTH levels change in response to experimental stress, differences between patients with rheumatic diseases (RA and SLE) and control groups have been reported in only two studies. There is preliminary evidence (one study) that the prolactin response to stress is different in patients than in controls.

#### Immune variables

Because several immune variables were used in the various studies to assess the effect of experimental stress on immune function, we have classified results on the basis of changes in leucocyte and lymphocyte counts and inflammatory markers (see Table 4).

##### Leucocytes and (subgroups of) lymphocytes

In patients with RA, experimental stress consistently increased the number of leucocytes [36,40,42], and increased the number of lymphocytes [30,40], with increases in the number of B cells [30], total T cells [40] and cytotoxic T cells [40] and either an increase [40] or a decrease [35] in helper T cells. The stress-induced increase in NK cell numbers [30,40] did not result in an increase in NK cell activity [40]. Most studies did not detect a difference in the immune response (leucocyte and lymphocyte counts) to stress of patients with RA and controls, but one study reported lower lymphocyte counts after stress in patients with RA [42]. Changes compared with controls were noted for helper T cells [35,40] and cytotoxic T cells [35]. In patients with SLE, stress induced a consistent increase in the number of leucocytes [40,43-45], and a less consistent increase in the number of lymphocytes [40,45]. Stress increased the number of total T cells [40,45], number [40,45] and percentage [43,44] of cytotoxic T cells, and the percentage of B cells [43], and decreased the number of helper T cells [35,43]. Again, although NK cell numbers increased after stress [40,45], NK cell activity did not increase [40,45]. Comparison showed that the immune response to stress was often smaller in patients with SLE than in controls: the increase in total leucocyte counts in patients with SLE was smaller than that of controls [40,43,45], as was the increase in total lymphocyte count [43-45]. Moreover, the stress-induced change in subsets of lymphocytes was often smaller for B cells [43,44], total T cell numbers [44,45], helper T cells [43-45], cytotoxic T cells [35,43], and NK cells [40,45].

Thus, total leucocyte counts and lymphocyte subsets change in response to stress in patients with rheumatic diseases, with patients with SLE having a smaller response (in addition to their baseline lymphopenia) than controls; no consistent alterations were found for patients with RA. NK cell cytotoxicity is diminished in patients with RA and SLE compared with control.

##### Inflammatory markers

In patients with RA, experimental stress did not change levels of various inflammatory markers (IL-2, soluble IL-2 receptor, IL-4, IL-10 and IFN-γ) [40,42], although levels of CRP [36] and TNF-α increased after stress [39,41]. Results for IL-6 were inconsistent, with stress inducing a decrease [41], an increase [39] or no change [40] in levels. Patients differed from controls, having smaller IL-10 and IFN-γ responses [40], and larger TNF-α [39,41] and CRP responses [36]. In patients with SLE, experimental stress did not change levels of cytokines and other inflammatory markers (IL-2, IL-6, IL-10, IFN-γ, β-adrenoceptors) [40,45], although levels of IL-4 increased after stress [40]. Patients with SLE differed from controls in their response to stress, having a larger increase in IL-4, smaller IL-10 and IFN-γ responses [40], and fewer β-adrenoceptors on monocytes [40,45]. In patients with JIA, stress did not affect IL-8 production, but increased IL-6 production [46]. This increase was not observed in controls. Thus, although in general experimental stress does not seem to influence levels of certain cytokines and inflammatory markers in patients with rheumatic diseases (for example, sIL-2, IL-8, IL-10, IFN-γ, β-adrenoceptors), it does cause specific changes in CRP and TNF-α in patients with RA, changes in IL-4 in patients with SLE, and changes in IL-6 in patients with JIA. These changes are not observed in controls.

In summary, the leucocyte and lymphocyte responses to stress are smaller in patients with SLE than in controls but consistent differences with controls are not seen in patients with RA. Experimental stress does not seem to affect NK cell cytotoxicity in either patient group. However, specific stress-induced changes in inflammatory markers are reported in patients with RA (CRP, TNF-α), SLE (IL-4) and JIA (IL-6) that are not observed in controls.

### Physiological stress reactivity for specific types of stressors

As seen above, experimental stress has a distinct effect on the autonomic, neuroendocrine, and immune systems. Below, we summarize the effect of the different types of experimental stress (cognitive, psychosocial, physical, and sensory), to determine whether different types of stress elicit different responses in different patients groups (see Table 5). Studies in which more than one type of stressor was used, were not included [38,42] unless outcome measures were reported separately for the different types of stressors [31,32].

**Table 5:** Autonomic (ANS), neuroendocrine (NE), and immune responses to different stressors in patients with systemic inflammatory rheumatic diseases

Stress paradigm	Studies	ANS		NE system		Immune system	
		*Measure*	*Response*	*Measure*	*Response*	*Measure*	*Response*
Psychological stress		HR	↑ RA[29,37]	Cortisol	↓/↑ RA[30]	Leucocytes	↑ RA[36]
			↑ Arthr[32]		0 SLE[44]		↑ SLE[44]
Cognitive tasks	[38]* [29,30,44]	SC	↑ RA[29]			Lymphocytes	↑ RA[30]
	[31]** [42]*		↑ Arthr[32]				0 SLE[44]
	[32,36,37]	CO	↑ RA[37]			T	0 RA[30]
		PV	↓ RA[36]				0 SLE[44]
		SVR	↑ RA[37]			B	↑ RA[30]
		BP	↑ RA[29,36,37]				0 SLE[44]
		CA	↑ SLE[44]			NK	↑ RA[30]
							0 SLE[44]
						Th	0 SLE[44]
						Tcyt	↑ SLE[44]
						CRP	↑ RA[36]

Psychosocial	[40,41,45]	HR	↑ RA[41]	Cortisol	↑ RA[41]	Leucocytes	↑ RA[40]
tasks			↑ SLE[45]	ACTH	↑ RA[41]		↑ SLE[40,45]
		BP	↑ RA[41]			Lymphocytes	↑ RA[40]
		PEP	↓ RA[41]				↑ SLE [40,45]
		CA	↑ RA[40]			T	↑ RA[40]
			↑ SLE[40,45]				↑ SLE[40,45]
						Tcyt	↑ RA[40]
							↑ SLE[40,45]
						NK	↑ RA[40]
							↑ SLE [40,45]
						Th	↑ RA[40]
							0 SLE[40,45]
						NK toxicity	0 RA[40]
							0 SLE[40,45]
						IL-6	↓ [41]/0 [40] RA
							0 SLE[40]
						IL-4	0 RA[40]
							↑ SLE[40]
						IL-2	0 RA[40]
							0 SLE[40]
						IL-10	0 RA[40]
							0 SLE[40]
						IFN-γ	0 RA[40]
							0 SLE[40]
						TNF-α	↑ RA[41]
						β - AR	0 SLE[41]

Exercise	[38]* [34,35]			Cortisol	↓ RA[34,35]	Th	↓ RA[35]
					↓ SLE[35]		↓ SLE[35]
				ACTH	↑ RA[34]	Tcyt	0 RA[35]
				GH	↑ RA[34]		↓ SLE[35]
				IGF-I	0 RA[34]		
				PRL	0 RA[35]		
					0 SLE[35]		

Sensory stress							
Cold induction	[38]* [39]	NE	↑ JIA[46]	Cortisol	↑ RA[39]	Leucocytes	↑ SLE[43]
	[31]** [46]					Lymphocytes	0 SLE[43]
	[33]**					T	0 SLE[43]
Heat induction	[39]					Tcyt	↑ SLE[43]
Acoustic stress	[43,42]*					B	↑ SLE[43]
Pupillary light	[32]**					Th	↓ SLE[43]
						IL-6	↑ RA[39]
							↑ JIA[46]
						TNF-α	↑ RA[39]

↑ = increase in response to stressor, ↓ = decrease in response to stressor, 0 = no response to stressor. RA = rheumatoid arthritis, SLE = systemic lupus erythematosus, JIA = juvenile idiopathic arthritis, arthr = heterogeneous group of patients with inflammatory arthritis. * = study not described in Table because more than one stress paradigm was used, ** = study not described in Table due to lack of within-subject measurements. HR = heart rate, SC = skin conductance, BP = blood pressure, NE = norepinephrine, CA = catecholamines, PC = pupillary constriction, PV = plasma volume, PEP = pre-ejection period, CO = cardiac output, SVR = systemic vascular resistance, C = cortisol, ACTH = adrenocorticotropin hormone, PRL = prolactin, GH = growth hormone, β-AR = β adrenoceptor, IGF-I = insulin-like growth factor I, IFN-γ = interferon-γ, TNF-α = tumor necrosis factor α, IL = interleukin, CRP = C-reactive protein, B = B cell, T = T cell, Th = helper T cell, Tcyt = cytotoxic T cell, NK = natural killer cell.

#### Cognitive stressors

Five studies investigated a cognitive stressor in patients with RA, SLE, or inflammatory arthritis [29-32,36,37,44]. In all patient groups, cognitive stress consistently increased autonomic function: heart rate [29,32,37], blood pressure [29,36,37], skin conductance [29,32], cardiac output and systemic vascular resistance [37], plasma volume [36], and catecholamines [44]. Patients with RA and SLE had different cortisol responses to cognitive stress [30,44] (see Table 5). Cognitive stress elicited changes in leucocyte counts, lymphocyte counts, subsets of lymphocytes, and CRP levels in patients with RA [30,36], but only changes in leucocyte counts and cytoxic T cell numbers in patients with SLE [44].

#### Psychosocial stressors

A psychosocial stressor was used in three studies of patients with RA and SLE [40,41,45]. Psychosocial stress consistently increased autonomic activity (heart rate [41,45], blood pressure [41], pre-ejection period [41] and catecholamine levels [40,45]) in patients with RA and SLE and increased neuroendocrine variables (cortisol and ACTH [41]) in patients with RA. Some immune responses were similar in the two patient groups (leucocyte counts, lymphocyte counts, (cytotoxic) T cells, NK cells and NK cell cytoxicity [40,45]), whereas others were different (helper T cells [40], IL-4 [40]) (see Table 5). Cytokine levels were not influenced by psychosocial stress, with the exception of an increase in IL-4 in patients with SLE, an increase in TNF-α in patients with RA, and inconsistent findings for IL-6.

#### Exercise stressors

The stress of exercise was investigated in two studies with patients with RA and SLE [34,35]. These studies did not assess autonomic function; however, cortisol levels decreased in both groups of patients [34,35] whereas ACTH levels and GH levels increased [34]. Exercise stress elicited a different cytotoxic T cell response in patients with RA and SLE [35] (see Table 5).

#### Sensory stressors

Sensory stressors were used in six studies involving patients with SLE and JIA [31-33,39,43,46]. Sensory stress increased catecholamine levels [46] and cortisol levels [39]. It also increased leucocyte counts, changed percentages of certain subsets of lymphocytes [43], and increased levels of IL-6 [39,46] and TNF-α [39] (see Table 5).

In summary, although few data are available about the effects of specific types of stress, results suggest that psychosocial, cognitive and sensory stress consistently increase autonomic activity. Changes in neuroendocrine variables in response to psychosocial, cognitive and sensory stressors are observed in patients with RA only. Psychosocial stress seems to elicit the strongest immune response in all patient groups studied.

## Discussion

A better understanding of the acute physiological response to stress in patients with inflammatory rheumatic diseases might further our knowledge of how stress affects the health of these patients. We reviewed the effects of time-limited stressors on autonomic, endocrine, and immune variables in patients with chronic inflammatory rheumatic diseases. Results suggest that autonomic and neuroendocrine responses to stress are not consistently altered in patients with rheumatic disorders compared with controls, although patients do appear to show a distinct immune response to stress. Psychosocial stress might prove to be the best tool to evaluate these specific immune responses to stress.

### Physiological stress response systems

#### ANS

Although previous studies that used regular autonomic function tests showed autonomic reactivity to be altered in different populations of patients with rheumatic diseases compared with controls [2,47-50], we failed to find consistent evidence for these alterations in studies investigating psychological, exercise, or sensory stress. The autonomic function of patients with rheumatic disorders was not only similar to that of controls at baseline but also after experimental stress, with cardiovascular, galvanic and catecholaminergic responses largely comparable to those of controls. Only a minority of studies reported autonomic function to be altered in these patients (as compared with controls) [29,31,32,37]. Studies have shown that alterations in autonomic function in response to stress are correlated with disease severity [36,37,48,51], which suggests that only subgroups of patients with severe disease show distinct alterations in autonomic function. Changes in autonomic function might also be linked to physical deconditioning, vascular inflammation and accelerated atherosclerosis [52], which could explain the inconsistencies in the literature on this subject. It should also be noted that alterations in autonomic function might reflect a change in sympathetic or parasympathetic activity or in the balance of the two systems [53,54]. Autonomic measures used in experimental stress paradigms (for example, the heart rate response) do not always differentiate between these systems, in contrast to regular autonomic function tests [55], which specifically aim to distinguish between sympathetic dysfunction and parasympathetic neuropathy [49,51,56].

#### HPA axis

The expected increase in neuroendocrine variables in response to a stressor was only observed in two studies with patients with RA [39,41]. This increase is consistent with previous studies reporting significant and consistent endocrine responses to psychosocial stress both in healthy subjects and in various patient populations without rheumatic disorders [24,25,41,57-62]. The change in cortisol levels is consistent with the endocrine response to real-life anticipation stress before hip or knee surgery in patients with RA [63,64]. However, most studies evaluated in this review reported a decrease in cortisol levels after stress, both in patients and controls, which might reflect the normal diurnal rhythmic decline in cortisol levels instead of a stress response [34,35,38,42]. All studies, except the two reporting an increase in cortisol levels, assessed patients in the early morning hours, when cortisol levels are known to decrease sharply.

Fuelling the ongoing debate on whether or not HPA axis responses are diminished in patients with rheumatic disease compared with healthy subjects, we did not find consistent changes in HPA axis function in response to psychological and exercise stress in patients with rheumatic diseases. Indeed, only two studies showed a different neuroendocrine response to stress in patients and controls [35,38]. Previous studies involving pharmacological stimulation of the HPA axis reported either similar or slightly lower responses in patients with rheumatic diseases compared with controls [27,28]. However, results should be interpreted with caution because stress manipulation may have been ineffective. Variations across studies in disease duration and activity, age, sex and sampling time might contribute to the variability in HPA axis responses in patients with rheumatic diseases. Moreover, attention should be paid to the role of pharmacotherapy (for example, corticosteroids), which could influence the endocrine response to stress [65].

#### Immune system

Most studies reported that total leucocyte counts and specific subsets of lymphocytes (not total number per se) increased after stress in patients with rheumatic diseases. The most consistent finding was an increase in the number of NK cells, as reported in earlier studies showing that the most robust effect of acute time-limited stressors in healthy subjects is a marked increase in the number of NK cells, which suggest an activation of innate immunity [15]. However, NK cell cytotoxicity after stress was lower in patients than in controls, possibly due to permanent activation as a consequence of the inflammatory disease [40,45]. The change in cytokine levels elicited by stress in patients with rheumatic disorders was small or inconsistent, for example, for the most frequently measured cytokine IL-6 [40,41,46]. Previous semi-experimental studies with patients with RA reported ambiguous results, reporting either significantly increased [63] or decreased levels of IL-6 [64] during anticipation of planned knee or hip arthroplasty. In addition, in a study evaluating responses to cryotherapy, patients with RA not treated with glucocorticoids responded with an increase in IL-6 levels [65]. A recent meta-analysis showed IL-6 levels to be consistently increased after psychosocial stress in healthy individuals [16]. The contradictory findings regarding cytokine responses in patients with rheumatic diseases might be due to methodological caveats, such as failed stress manipulation or small sample sizes (for example, in Jacobs et al. (2001)). However, it is also possible that cytokines do not effectively regulate immune function systemically, but instead near the effector site [40]. Future studies involving patients with rheumatic diseases should measure local immune variables in samples obtained from joint tissue or synovial fluid [66,67]. Another explanation is that patients or subgroups of patients might have different cytokine responses (for example, TNF-α) because of differences in disease severity [68] or pharmacotherapy [41,65]. In general, as could be expected for immune-mediated diseases, the immune response to stress was different in patients and controls, and also differed between the various types of inflammatory rheumatic diseases.

### Differences in type of stressor, rheumatic disease and (time points of) outcome measures

Findings of this review should be interpreted with caution due to differences in the types of stress induced, the various rheumatic diseases evaluated, and the variability in outcome measures and assessment times. In addition, the highly variable methodological quality of the studies, the small number of studies, and the relatively small patient samples hinder an unequivocal interpretation of findings. Future research urgently requires well-designed studies with larger numbers of patients. These studies should, for example, systematically take into account physiological baseline levels.

#### Stress paradigms

Only the studies of psychosocial stress evaluated all three physiological stress response systems (autonomic, neuroendocrine and immune) and, moreover, consistently reported stress-induced increases at all three levels. This is in line with previous studies showing significant and consistent autonomic, neuroendocrine, and immune responses to psychosocial stress in both healthy subjects and patient groups [25,41,57-62,69]. In contrast, cognitive stressors did not consistently alter neuroendocrine variables, and immune responses were small in patients with SLE. The stress of exercise was found not to activate the HPA axis in patients with rheumatic diseases, in contrast to what has previously been observed in healthy subjects [70], although studies suggest that neuroendocrine changes are only observed after exercise of longer duration [71]. Data on immune responses were very limited. The studies that assessed sensory stress reported stress-induced increases in autonomic, neuroendocrine, and immune function, but data were limited. Because most sensory stressors are also frequently applied to induce pain [72,73], future studies of stress should take into account whether a stressor is pain-inducing in order to unravel the relationship between stressors, pain and physiological responses [74]. Overall, more research into the effects of psychosocial stress on physiological function is needed, especially because these stress paradigms seem to generate the strongest response at all three physiological levels and probably have the greatest ecological validity. Importantly, insight in physiological responses to acute stressors cannot automatically be translated to the field of daily stressors, major stress events or even chronic stressors [5,75]. For example, studies in healthy and other disease populations suggest that chronic stress might induce hypocortisolism [6,76] and global immunosuppression of both innate and specific immunity [15]. Overall, there is a lack of studies examining the association between physiological parameters and chronic stressors in rheumatic populations [2]. Studies investigating stressors in daily life reported an association between stressors, mainly of an interpersonal nature, and concentrations of cytokines and lymphocytes (for example, IL-6, soluble IL-2 receptors and T cell numbers) [77-79]. However, results are inconclusive [80] and future studies are needed to elucidate how acute and chronic stressors differentially affect inflammatory rheumatic diseases.

#### Disease and treatment characteristics

In contrast to autonomic and neuroendocrine responses, the immune response elicited seemed to be specific to the type of inflammatory rheumatic disorder involved, and this was especially evident for psychosocial stress. Patients with SLE often have baseline lymphopenia and this review showed that stress led to diminished cell mobilization and consequently less pronounced stress-induced changes in lymphocyte (sub)populations in these patients compared with controls and patients with RA. In contrast, patients with RA did not consistently show these alterations, but only alterations in certain subpopulations of lymphocytes. Furthermore, the cytokine response to stress might be distinct for different rheumatic populations. The effect of pharmacotherapy (for example, corticosteroids and biologicals) on neuroendocrine and immune responses to stress in these populations is unclear. Only two of 16 studies conducted subgroup analyses to reveal possible effects of pharmacotherapy on response patterns. Motivala et al. (2008) found significant stress-induced increases in TNF-α only in patients with RA not treated with TNF-α antagonists (and no effect of corticosteroid treatment), and Pawlak et al. (1999) reported no significant difference in physiological variables between patients with SLE treated with corticosteroids and/or disease-modifying antirheumatic drugs (DMARDs) and non-treated patients [45]. Four other studies also suggest no effect of pharmacotherapy (more specifically, DMARDs [38], nonsteroidal anti-inflammatory drugs (NSAIDs) [34,38], and corticosteroids [34,43,44]) on stress reactivity patterns based on descriptive data. The other studies do not mention possible effects of pharmacotherapy [29-33,35-37,39,40,42,46].

Clearly, patient samples are often too small to draw conclusions. More studies comparing different inflammatory diseases and healthy participant are needed to address the question whether the physiological response to stress is disease-specific, knowledge which might facilitate our understanding of factors contributing to the maintenance and exacerbation of rheumatic disorders.

#### Outcome measures

The heterogeneity of outcome measures, especially with regard to immune variables, makes it difficult to compare studies of the stress response. Moreover, stressors that activate the physiological stress system via the hypothalamus and subsequent down-stream cascades might induce alterations in hormones, peptides and cytokines other than discussed and assessed in this review (for examples, dehydroepiandosterone (sulfate) (DHEA-S), neuropeptide Y (NPY), substance P). Previous studies suggest that alterations in the stress response in patients with rheumatic disorders might be located in the interaction between the two stress axes (HPA and ANS) and the immune system, more specifically in the number or signalling capacity of β-adrenoceptors [45,48,66,81] or glucocorticoid receptors [82-84] on lymphocytes.

Although the HPA axis, ANS, and immune system are thought to function in an interdependent fashion, only four of 16 studies in this review investigated outcome variables that evaluate all three stress response systems. Cardiovascular reactivity during a mental stress task has been shown to be associated with the subsequent cortisol response [25], and cardiovascular responses have been associated with post stress levels of cytokines such as IL-6 and TNF-α [85]. In addition, HPA axis variables are correlated with immune responses after stress [53]. Future studies should try to integrate the responses of different systems by simultaneously assessing (para)sympathetic responses, neuroendocrine variables, and immune factors, to increase our knowledge of the coordination and possible dysregulation of these systems [59].

#### Psychological variables

In addition to assessing the physiological response to experimental real-life stressors, it is important to consider other key elements of the stress response, such as the individual's appraisal of the threat of the event, perceived distress, coping behaviour and personality characteristics [4]. As the appraisal of threat might partly determine the biological stress response of an individual, self-reported measures of distress appear to be a simple and adequate manipulation check in studies of psychosocial stress. Furthermore, acute stress responses are known to be correlated with individual differences in psychological factors, such as coping styles [86], trait anxiety [87,88], depressed mood [59,89,90], perfectionism [91], social inhibition [92] and anticipatory cognitive appraisal [93-96]. However, none of the studies, except one [74], assessed individual psychological characteristics. In addition, only two studies in this review assessed stress-induced self-reported distress [41,42] and only three studies [36,37,39,41] explicitly excluded affective disorders. Although half of all studies excluded patients with (severe or chronic) comorbidity, these were mainly physical in nature. We cannot rule out the possibility that patients with psychiatric disorders are included in these studies, obscuring the major research question whether inflammatory rheumatic diseases are related to a specific physiological stress responses profile. Future studies of stress responses in patients with rheumatic diseases should include individual psychological characteristics (for example, personality, coping styles) and affective responses to help identify whether patients differ with regard to psychophysiological responses to stress.

#### Time points

Some consideration should be given to when outcomes are measured. Almost all the studies included at least one immediate post stress measurement, but only half of the studies included an additional measurement (mostly 30 or 60 minutes after cessation of the stressor), with only two studies including more than two follow-up measurements. Cytokine responses may be delayed for minutes or hours relative to ANS and HPA responses, because cytokines are produced by activated lymphocytes and macrophages [97-99]. This makes it difficult to detect the peak immune response when collecting only one or two samples, for example for the increase in IL-6 and IL-1 receptor agonists following psychosocial stress [85].

Additionally, in accordance with the allostatic load concept [100] the ability and time taken by the activated stress system to return to baseline (recovery period) might be an important factor in the link between stress and disease [99,101-103]. Moreover, repeated mental stress has been linked to a lack of habituation of the cortisol response [89,104] and plasma interleukin-6 [99] in subgroups of individuals. Thus future studies should have more frequent evaluation time points and investigate individual differences in the return-to-baseline values and in habituation patterns after repeated stress.

## Conclusions

In summary, this review shows that there is limited evidence that autonomic and neuroendocrine function is altered after physical or psychological stress in patients with inflammatory rheumatic diseases compared with healthy subjects. In contrast, there is evidence that immune function is altered by stress in a manner specific to different rheumatic diseases, and thus real-life stressors could contribute to the maintenance or exacerbation of rheumatic diseases.

Future studies of stress, and particularly psychosocial stress, should have a follow-up with multiple measurement points, assess different physiological stress systems, and take into account stress appraisal. As individual differences in stress appraisal and stress response patterns might be important prognostic factors for disease progression, therapies that focus on stress management may be important adjuncts to traditional pharmacotherapy in the treatment of inflammatory rheumatic diseases. Stress induction studies could prove to be helpful for evaluating the effectiveness of these interventions [105,106].

## Abbreviations

ACTH: adrenocorticotropin hormone; ANS: autonomic nervous system; CNS: central nervous system; CRH: corticotropin-releasing hormone; CRP: C-reactive protein; DHEA(-S): dehydroepiandosterone (sulfate); DMARDs: disease-modifying antirheumatic drugs; GH: growth hormone; HPA axis: hypothalamus-pituitary-adrenal axis; IFN-γ: interferon-γ; IGF-I: insulin-like growth factor I; IL: interleukin; sIL-2 receptor: soluble interleukin-2 receptor; JIA: juvenile idiopathic arthritis; NK: natural killer cell; NPY: neuropeptide Y; NSAIDs: nonsteroidal anti-inflammatory drugs; PEP: pre-ejection period; RA; rheumatoid arthritis; SLE: systemic lupus erythematosus;  TNF-α: tumor necrosis factor α.

## Competing interests

The authors declare that they have no competing interests.

## Authors' contributions

SdB and AE drafted the manuscript. The other authors critically revised it and approved the final content of the manuscript.
